# Preserving the antimicrobial arsenal: exploring alternatives to carbapenems in ESBL battles within the southeast of Ireland

**DOI:** 10.1099/jmm.0.001955

**Published:** 2025-02-05

**Authors:** Saied Ali, Aideen Tobin, Susan Lapthorne, Meadhbh Collison, Doireann Murphy, Grace Chan, Maeve Doyle

**Affiliations:** 1 Department of Clinical Microbiology, University Hospital Waterford, Waterford, Ireland; 2 Royal College of Surgeons in Ireland, Dublin, Ireland

**Keywords:** antimicrobial resistance, antimicrobial stewardship, antimicrobials, carbapenems, *Enterobacterales*, ESBL, extended-spectrum beta-lactamase, surveillance

## Abstract

**Introduction.** Carbapenems are usually employed as first-line antimicrobials against bacteria harbouring extended-spectrum beta-lactamases (ESBLs). These enzymes confer resistance often to multiple classes of antimicrobials.

**Hypothesis/Gap Statement.** This indiscriminate use of carbapenems and the inevitable development of carbapenem resistance have prompted the need for carbapenem-sparing strategies.

**Methodology.** The non-carbapenem antimicrobial susceptibility patterns of 60 ESBL-producing *Enterobacterales* (ESBL-PE) isolates responsible for bloodstream infections, in 2022–2023 inclusive, processed at our institution were reviewed.

**Results.** The non-carbapenem antimicrobial susceptibility patterns of 60 ESBL-PE isolates from bloodstream infections during the study period were determined. *Escherichia coli* was the most common species isolated (87%, *n*=52), with the majority of cases (73.3%, *n*=44) originating from a presumed urinary source. Temocillin (TMC), mecillinam (MEC), cefiderocol (FDC), amikacin and fosfomycin (FOS) displayed excellent activity against all ESBL-PE isolates tested, with susceptibility rates of≥85%. Ciprofloxacin and amoxicillin–clavulanic acid were the least efficacious agents, with susceptibility rates≤20%.

**Conclusions.** TMC, MEC, FDC and FOS offer promising alternatives to carbapenems, demonstrating efficacy against ESBL-PE. The use of these agents not only broadens the therapeutic arsenal against ESBL-PE but also mitigates the potential for escalating carbapenem resistance, especially in regions where the incidence of carbapenem resistance is increasing.

## Data Summary

The authors confirm all supporting data, code and protocols have been provided within the article.

## Introduction

Bacteria harbouring extended-spectrum beta-lactamases (ESBLs), especially within the *Enterobacterales* family, represent a significant challenge in healthcare due to their resistance to a wide range of beta-lactam antimicrobials. These enzymes enable hydrolysis of penicillins, cephalosporins and monobactams, often rendering standard beta-lactam therapies ineffective [1,2]. This issue is exacerbated when ESBLs co-exist with other resistance genes, such as those conferring resistance to fluoroquinolones (FQs) and aminoglycosides (AGs) like aac(6′)-Ib-cr and qnr and armA, respectively, resulting in multidrug-resistant (MDR) ESBL-producing strains [3,5]. As a result, carbapenems have become the cornerstone of treatment due to their reliable efficacy against these pathogens [1].

However, the increasing reliance on carbapenems has contributed to the emergence of carbapenem-resistant organisms, narrowing the already-limited therapeutic options. The incidence of bloodstream infections due to carbapenem-resistant *Enterobacterales* has increased by 50% from 2019 to 2023 across the European Union/European Economic Area (EU/EEA) [6]. This highlights the critical need for alternative, carbapenem-sparing strategies to preserve the effectiveness of current antimicrobials [5].

A targeted approach in identifying effective alternatives to carbapenems reduces reliance on these broad-spectrum agents, minimizing selective pressure on *Enterobacterales*. From a global antimicrobial stewardship (AMS) perspective, advancing antimicrobial susceptibility testing for carbapenem-sparing agents fosters equitable access to tailored therapies, supports sustainable healthcare practices and enhances the capacity to combat antimicrobial resistance (AMR), ultimately protecting global health security [7,8].

This study aimed to evaluate the antimicrobial susceptibility profiles of ESBL-producing *Enterobacterales* (ESBL-PE) isolates from bloodstream infections in the southeast of Ireland. The study focused on potential carbapenem-sparing options, including amoxicillin–clavulanic acid (AMC), piperacillin–tazobactam (TZP), temocillin (TMC), mecillinam (MEC), cefiderocol (FDC), ciprofloxacin (CIP), gentamicin (GEN), amikacin (AMK), eravacycline (ERV), fosfomycin (FOS) and trimethoprim–sulfamethoxazole (SXT).

## Methods

An analysis of the non-carbapenem antimicrobial susceptibility patterns of 60 ESBL-PE isolates from bloodstream infections, collected between 1 January 2022 and 31 December 2023 inclusive, processed at University Hospital Waterford (UHW), was performed. All ESBL-PE isolates cultured from blood during the study period were reviewed, with only one isolate per patient included when multiple sets of blood cultures from that patient yielded the same bacterial species.

UHW is a 500-bedded academic teaching hospital in the southeast of Ireland, serving as the regional hub for acute, chronic and emergency care. It supports nearby Model Three hospitals – St Luke’s General Hospital Kilkenny, Wexford General Hospital and Tipperary University Hospital [9]. All positive blood cultures from these hospitals are processed at UHW, ensuring a representative sample for the region.

ESBL screening was performed on all *Enterobacterales* isolates using the MASTDISCS® Combi Cefpodoxime ESBL ID Disc Set (Mast Group, Bootle, Merseyside, UK). The presence of ESBL-PE was confirmed when a difference of≥5 mm was observed between the inhibition zones of the cefpodoxime-only disc and the combination disc plus clavulanic acid, as per the manufacturer’s guidelines [10,12]. Cefpodoxime is a third-generation cephalosporin used as an ESBL screening agent due to its sensitivity to hydrolysis by ESBLs. Its standardized breakpoints and ease of use in confirmatory tests with beta-lactamase inhibitors, such as clavulanic acid, ensure reliable detection of ESBL production [12,13]. A quality control strain, *Escherichia coli ATCC 25922*, was used to validate screening procedures.

Antimicrobial susceptibility testing (AST) was conducted following the European Committee on Antimicrobial Susceptibility Testing (EUCAST) guidelines. The disc diffusion methodology [14] was used with Becton Dickinson (BBL™) discs for AMC, TZP, CIP, GEN, AMK, FOS and SXT and MASTDISCS (Mast Group) discs for FDC. MICs were determined using gradient strips (E-tests, bioMérieux) for TMC, MEC and ERV, following manufacturer instructions. All zone diameters and MIC values were manually measured and interpreted according to EUCAST breakpoint tables (Version 14) [15]. Due to available resources, duplicate testing was not possible. However, results were deemed reliable once quality control strains met the required performance standards.

These antimicrobials were chosen based on regional availability, demonstrated clinical success in neighbouring institutions as effective carbapenem-sparing agents and the availability of established EUCAST AST methodologies and breakpoints.

Descriptive statistics were employed to succinctly summarize and present the findings of the study.

## Results

ESBL-PE accounted for 35 bloodstream infections in 2022 and 37 in 2023. Of the 72 isolates collected, 12 were unavailable for analysis due to missing data and 60 were analysed, with 25 cases (41.7%) linked to previously known colonization and 35 (58.3%) newly identified. The urinary tract was the presumed source of the bacteraemia in most cases (73.3%, *n*=44), followed by intra-abdominal (18.3%, *n*=11), bone and joint (3.3, *n*=2), skin and soft tissue (1.7, *n*=1) and unknown (3.3%, *n*=2). All sources of infection were determined by clinical assessment. There were no mixed sources of infection considered. Meropenem (MEM) was used as definitive therapy in 93.3% (*n*=56) of cases.

**Fig. 1.**
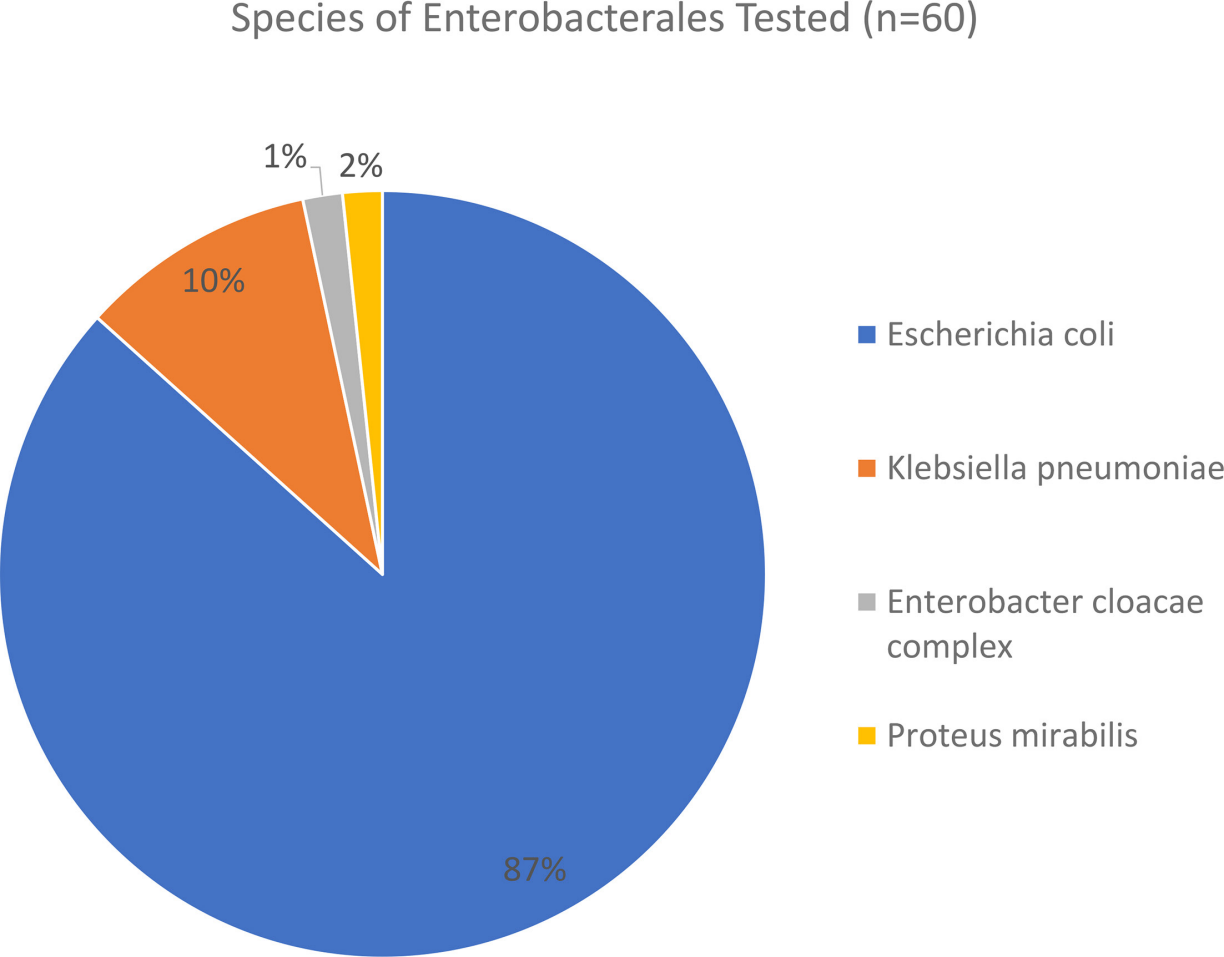
Species of *Enterobacterales* reviewed.

*E. coli* was the predominant species isolated (87%, *n*=52), as shown in Fig. 1.

**Fig. 2.**
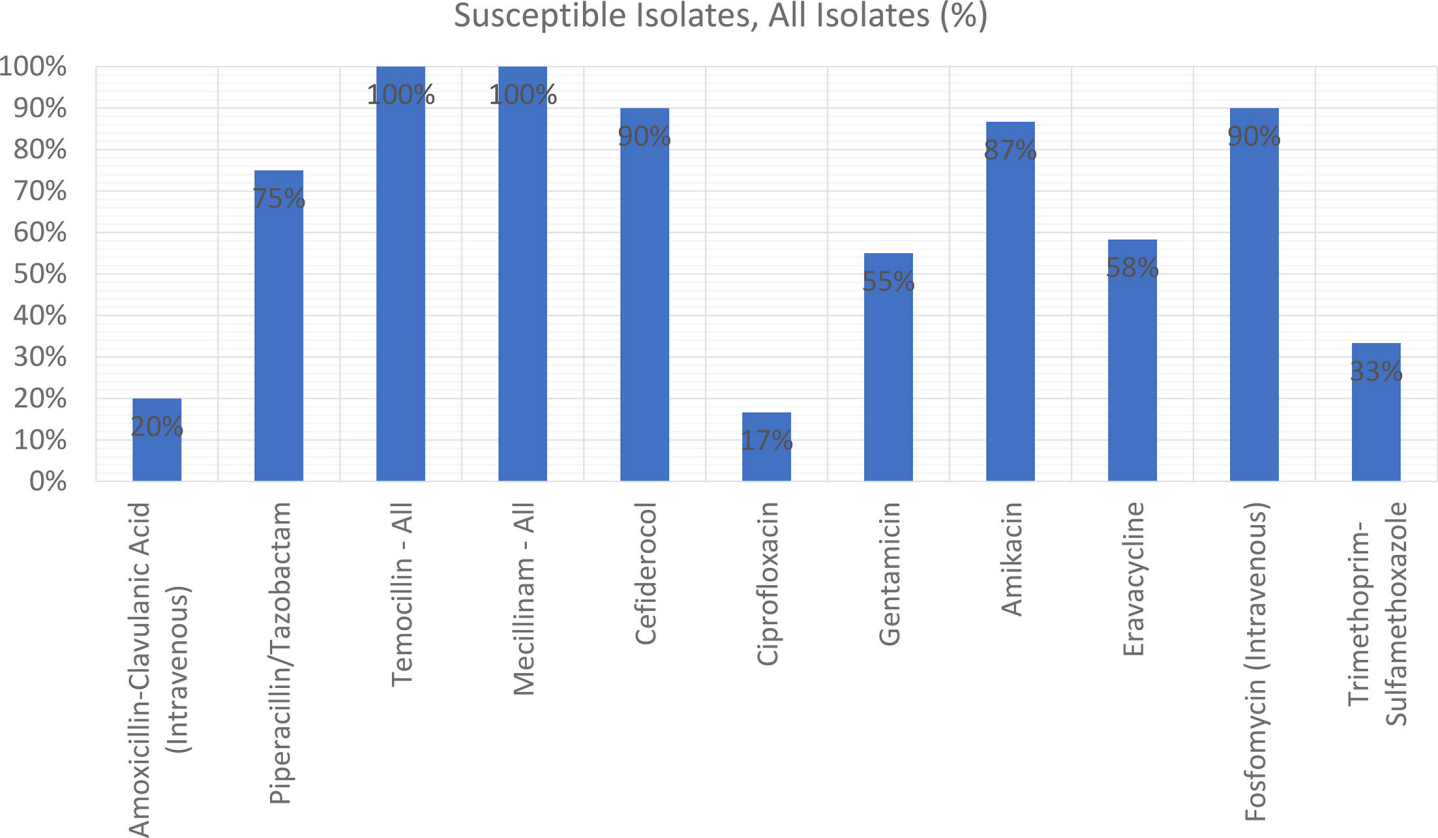
Percentage of susceptible isolates among all isolates to antimicrobials tested. All susceptibility results were interpreted according to EUCAST breakpoints. In cases where specific breakpoints are not well-defined, such as for certain bacterial species or sources of infection, susceptibility was interpreted based on *Enterobacterales* breakpoints, disregarding species-specific and source-specific restrictions.

Susceptibility patterns (Fig. 2 and Table 1) revealed that TMC, MEC, FDC, AMK and FOS displayed excellent activity against the ESBL-PE tested, with susceptibility rates of≥85%.

**Table 1.** Percentage of resistant isolates to antimicrobials tested

	AMR rate, %										
	AMK	AMC (Intravenous)	FDC	CIP	ERV	FOS (Intravenous)	GEN	MEC	TZP	TMC	SXT
*Overall resistance rate (n=60*)	13	80	10	83	42	10	45	0	25	0	67
*E. coli only (n=52*)	13	79	12	85	37	0	46	0	17	0	67
*Non-E. coli (n=8*)	13	88	0	75	75	75	38	0	75	0	63
*Urinary sources – All (n=44*)	11	77	7	86	32	9	48	0	25	0	68
*Urinary sources – E. coli only (n=40*)	10	78	8	88	28	0	48	0	20	0	65

**Fig. 3.**
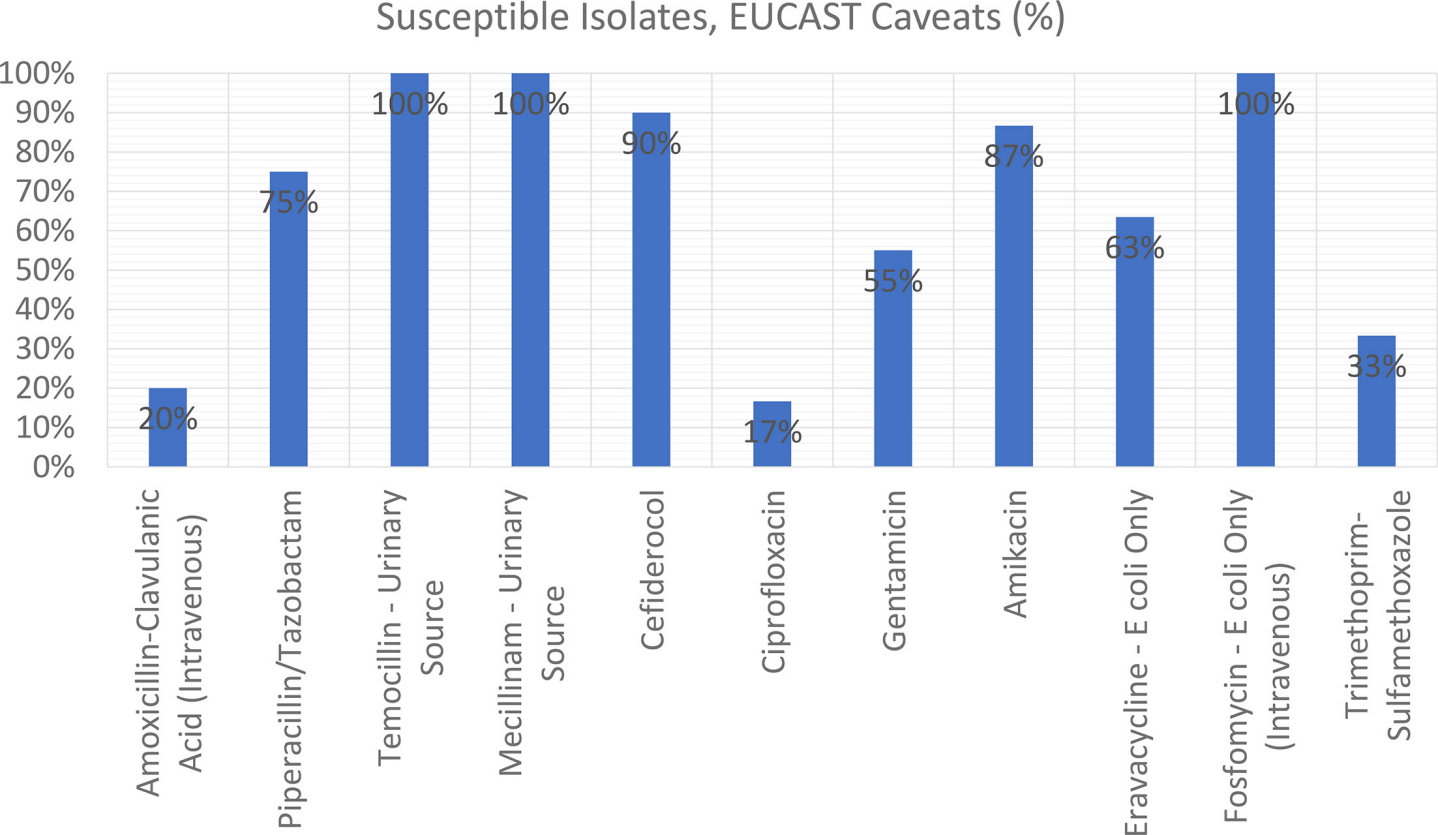
Percentage of susceptible isolates to antimicrobials tested with EUCAST caveats applied.

Clinical breakpoints for TMC, MEC and FOS are restricted to specific conditions per EUCAST guidelines. Breakpoints are only established for infections presumed to originate from the urinary tract and only for *E. coli* in the case of FOS; *E. coli*, *Klebsiella* spp. (except *Klebsiella aerogenes*) and *Proteus mirabilis* for TMC; and *E. coli*, *Citrobacter* spp., *Klebsiella* spp., *Raoultella* spp., *Enterobacter* spp. and *P. mirabilis* for MEC. When these conditions were applied, as shown in Fig. 3 and Table 1, all applicable isolates showed 100% susceptibility; however, the relatively small number of tested isolates does limit the generalizability of these findings.

Conversely, CIP and AMC exhibited low efficacy, with susceptibility rates≤20%. SXT also performed poorly (33% susceptibility). These findings indicate limited utility for these agents in managing ESBL-PE bacteraemia and that choice of these agents should be guided by AST. Results also underscore the importance of drug development, as now oral therapies are further limited by AMR.

## Discussion

The European Centre for Disease Prevention and Control estimated over 800 000 antimicrobial-resistant infections in the EU/EEA in 2020, causing 35 000 deaths, with a projected global death toll of 10 million by 2050 [6].

Irish epidemiological data from 2018 to 2022 show a 6% rise in invasive infections caused by *Enterobacterales*, but a decrease in AMR, attributed to the effectiveness of enhanced AMS programmes and Ireland’s One Health National Action Plan. Notably, invasive infections due to ESBL-PE dropped from 11.6 to 9.1%, with similar decreases in AG and FQ resistance, while carbapenem resistance remained<1% [16,18].

Guidelines published by the European Society of Clinical Microbiology and Infectious Diseases inform antimicrobial prescribing practices in our region, which likely accounts for the high use (93.3%) of MEM as definitive therapy against ESBL-PE bloodstream infections over the study period. For the treatment of infections caused by MDR Gram-negative bacilli, carbapenems are recommended for severe infections caused by third-generation cephalosporin-resistant *Enterobacterales*, with alternatives, such as beta-lactam/beta-lactamase inhibitors (BLBLIs), SXT and FQs for low-risk cases [19]. The Infectious Diseases Society of America has similar guidance but is less inclined to encourage the use of BLBLIs due to findings from the MERINO trial [20,21].

Debate continues to surround the use of BLBLIs versus carbapenems for infections caused by ESBL-PE. The MERINO trial reported higher mortality rates for TZP compared to MEM for bloodstream infections caused by ceftriaxone-resistant *Enterobacterales* [21]. However, subsequent analyses showed less significant differences when focusing on ESBL-producing strains specifically and excluding strains resistant to TZP *in vitro*. More recently, a meta-analysis including one randomized controlled trial and 25 cohort studies concluded that BLBLIs were as effective as carbapenems for treating bloodstream infections caused by ESBL-PE [22]. Our findings suggest BLBLIs represent a potential carbapenem-sparing strategy, with 75% of our ESBL-PE isolates displaying *in vitro* susceptibility to TZP. However, with the conflicting literature, future *in vivo* studies remain warranted.

In our region, AGs are often recommended in combination therapy with either a BLBLI or carbapenem, with AMK being the AG of choice for life-threatening sepsis or septic shock. Year 2022 data from the European Antimicrobial Resistance Surveillance Network reported an overall AG resistance rate of 9.1% among Irish isolates [16]. Studies from Israel, Iran, Oman, the United States of America (USA) and Europe have all demonstrated reliable *in vitro* efficacy of AGs against ESBL-PE, with a notably greater proportion of isolates displaying GEN resistance when compared to AMK, 61.8% versus 4.8% [23,27]. This observation was also noted amongst our isolates, with non-susceptibility to GEN noted at 45% versus 13% for AMK, validating our choice of AMK for significant infection. However, the use of AGs is not without risk, particularly that of nephrotoxicity and ototoxicity, which in turn may favour alternative carbapenem-sparing options [23].

The rising global resistance to FQs and SXT limits their empirical use in treating infections caused by ESBL producers. FQ resistance rates have been reported to be as high as 82.2% in Europe, 70.9% in Taiwan, 65.2% in Iran, 56.8% in Brazil and 46% in the USA, and>30% globally for SXT [3,24, 28,32]. These figures mirror our study’s findings, with 83 and 67% non-susceptibility to CIP and SXT, respectively.

Our study also supports FOS’s potent antimicrobial activity against ESBL-PE, as 100% of the ESBL-producing *E. coli* isolates tested were susceptible to it. In randomized controlled trials (ZEUS and FOREST), intravenous FOS was compared with TZP and MEM, respectively. ZEUS included patients with complicated urinary tract infections, pyelonephritis and bloodstream infections, while FOREST focused on *E. coli* bacteraemia presumed to originate from urinary sources. Both trials found no significant difference in clinical or microbiological cure rates between FOS and comparators [33].

FDC, a novel cephalosporin with a unique structure, combines a siderophore and a beta-lactam base, making it resistant to hydrolysis by various beta-lactamases, including ESBLs. Following the success of the CREDIBLE-CR and APEKS studies, FDC has been approved for the treatment of infections caused by aerobic Gram-negative organisms [34]. Studies in Germany and the United Kingdom (UK) demonstrated potent activity against MDR *Enterobacterales*, where FDC inhibited over 80% of *Enterobacterales* isolates [35,37]. In total, 90% of isolates were inhibited in the current study. FDC is generally reserved for the treatment of infections caused by carbapenem-resistant or carbapenemase-producing organisms, so while it displays excellent activity against ESBL-PE, it may be prudent to conserve FDC for more extensively drug-resistant organisms [34].

TMC, a semi-synthetic 6-*α*-methoxy derivative of ticarcillin, is a narrow-spectrum beta-lactam antimicrobial effective against many Gram-negative bacteria, including those carrying ESBLs [38]. EUCAST has established clinical breakpoints for TMC for a limited number of bacterial species – *E. coli*, *Klebsiella* species (excluding *K. aerogenes*) and *P. mirabilis* – and only for urinary tract infections due to pharmacokinetic and pharmacodynamic properties. Strains with an MIC≤16 mg l^−1^ are categorized as ‘susceptible, increased exposure (I)’, meaning a posology of 2 g 8 h is required [15]. In the MERINO trial, 95% of bloodstream isolates had MIC values≤16 mg l^−1^ [39]. In various European studies, TMC showed potent activity against ESBL-PE, with susceptibility rates ranging from 62 to 99% [40,43]. All of our study isolates, of which 98% were species for which EUCAST has established breakpoints [15], were susceptible to TMC. Though promising for intra-abdominal infections, more research is needed to confirm TMC’s efficacy compared to other antimicrobials, especially due to the polymicrobial nature of abdominal infections and challenges in achieving therapeutic drug levels in peritoneal or intra-abdominal tissues. While Pfeiffer and Fock demonstrated its effectiveness in a small number of critically ill patients with peritonitis and intra-abdominal abscesses, from whom a variety of *Enterobacterales*, *Pseudomonas* and anaerobes were isolated, further studies are required to establish its non-inferiority [44].

MEC, particularly its oral prodrug pivmecillinam (PIV), has been widely used for decades in Scandinavian countries due to sustained low resistance rates in the community, around 5% [45]. An American study reported an overall MEC susceptibility of 95% against 986 ESBL-PE, while a Norwegian study found that PIV, given for 1 week after 3 days of parenteral antimicrobials, effectively treated *E. coli* bacteraemia presumed to originate from urinary sources among 49 non-critically ill patients [45,46]. A similar strategy may be considered locally, as all tested applicable isolates were susceptible to MEC. However, further studies remain warranted to strengthen the clinical evidence.

ERV, a novel fluorocycline antimicrobial, shows effectiveness against MDR organisms (MDROs), including ESBL-PE and carbapenem-resistant *Enterobacterales*. However, its approval by the USA Food and Drug Administration and the European Medicines Agency is currently limited to treating complicated intra-abdominal infections, based on IGNITE-1 and IGNITE-4 trial results. While IGNITE-2 and IGNITE-3 aimed to compare their efficacy and safety versus levofloxacin and ertapenem for the treatment of complicated urinary tract infections, they failed to prove non-inferiority [47]. Yet, global susceptibility testing of 10 531 *Enterobacterales* strains showed high rates of susceptibility to ERV, ranging from 89.6 to 98.8% across different species [48]. Similar findings from the USA and China confirm its potent activity against common and clinically significant MDROs [49,51]. With a susceptibility rate significantly lower than that of the abovementioned studies at only 58% among our isolates, further investigation remains warranted, especially for that of heteroresistance [52]. So, while ERV may represent a potential carbapenem-sparing strategy, AST remains important to support its use.

We recognize several limitations in our study. Our findings are based on a small cohort from a small geographic region, which limits their global applicability. AST was performed by disc diffusion or gradient-strip MIC determination as outlined in the Methods; this is not the gold standard method. The reference method for MEC and FOS is agar dilution, and that for FDC is broth microdilution. Nonetheless, our methodology has been validated by numerous previous studies producing reliable findings and endorsed by EUCAST [14]. ESBL detection was performed solely by inhibitory combination discs, and further molecular studies remain warranted to assess the nature of particular ESBL strains and the presence of co-resistance mechanisms.

## Conclusion

TMC, MEC, FDC and FOS exhibit promising potential as alternatives to carbapenems, showing significant efficacy against ESBL-PE. Their integration into treatment protocols can broaden therapeutic options, reserving carbapenems for more critical infections and helping to mitigate the growing issue of carbapenem resistance. However, it is crucial to conserve these novel agents for use in more extensively drug-resistant organisms to prevent the development of resistance. Continued research, including randomized controlled trials and long-term studies, is essential to solidify the role of these agents and to identify the most effective strategies for their use in clinical practice. These efforts are vital in the global fight against AMR, a pressing public health challenge that demands ongoing innovation and careful stewardship.

## Abbreviations

AGs: aminoglycosides
AMC: amoxicillin–clavulanic acid
AMK: amikacin
AMR: antimicrobial resistance
AMS: antimicrobial stewardship
AST: antimicrobial susceptibility testing
BLBLIs: beta-lactam/beta-lactamase inhibitors
CIP: ciprofloxacin
ERV: eravacycline
ESBL-PE: ESBL-producing *Enterobacterales*
ESBLs: extended-spectrum beta-lactamases
EUCAST: European Committee on Antimicrobial Susceptibility Testing
EU/EEA: European Union/European Economic Area
FDC: cefiderocol
FOS: fosfomycin
FQs: fluoroquinolones
GEN: gentamicin
MDR: multidrug-resistant
MDROs: multidrug-resistant organisms
MEC: mecillinam
MEM: meropenem
PIV: pivmecillinam
SXT: trimethoprim–sulfamethoxazole
TMC: temocillin
TZP: piperacillin–tazobactam
UHW: University Hospital Waterford
